# Functional analysis of human intrafusal fiber innervation by human γ-motoneurons

**DOI:** 10.1038/s41598-017-17382-2

**Published:** 2017-12-08

**Authors:** A. Colón, X. Guo, N. Akanda, Y. Cai, J. J. Hickman

**Affiliations:** Hybrid Systems Lab, NanoScience Technology Center, University of Central Florida, 12424 Research Parkway, Suite 400, Orlando, FL 32826 USA

## Abstract

Investigation of neuromuscular deficits and diseases such as SMA, as well as for next generation prosthetics, utilizing *in vitro* phenotypic models would benefit from the development of a functional neuromuscular reflex arc. The neuromuscular reflex arc is the system that integrates the proprioceptive information for muscle length and activity (sensory afferent), to modify motoneuron output to achieve graded muscle contraction (actuation efferent). The sensory portion of the arc is composed of proprioceptive sensory neurons and the muscle spindle, which is embedded in the muscle tissue and composed of intrafusal fibers. The gamma motoneurons (γ-MNs) that innervate these fibers regulate the intrafusal fiber’s stretch so that they retain proper tension and sensitivity during muscle contraction or relaxation. This mechanism is in place to maintain the sensitivity of proprioception during dynamic muscle activity and to prevent muscular damage. In this study, a co-culture system was developed for innervation of intrafusal fibers by human γ-MNs and demonstrated by morphological and immunocytochemical analysis, then validated by functional electrophysiological evaluation. This human-based fusimotor model and its incorporation into the reflex arc allows for a more accurate recapitulation of neuromuscular function for applications in disease investigations, drug discovery, prosthetic design and neuropathic pain investigations.

## Introduction

There has been a recent emphasis on the development of *in vitro* “human-on-a-chip” systems for use in drug discovery studies as well as basic cell biology investigations. While animal models have been the standard for disease and drug evaluation, many of the effects seen in these models are incongruent with the effects seen in humans. Furthermore, various organizations have restricted the use of animal models for ethical considerations. To avoid these difficulties, *in vitro* systems that integrate human cell culture with BioMEMs devices are being investigated for their ability to recapitulate functional human organ systems^1–8^. The high content analysis capabilities, reproducibility, ethical considerations, and biological flexibility with relevant cell types have increased the demand for realistic functional *in vitro* systems. The neuromuscular reflex arc is a highly complex biological circuit, where the actuation segment has been successfully evaluated using *in vitro* systems^9–14^. We have previously shown functional neuromuscular junction formation *in vitro* for both animal and human cells on a non-biological substrate^9,14^. We have also demonstrated the fundamental sensory portion of the arc by animal and human sensory neuron innervation of intrafusal fibers^12,15^. Further elaboration of this system, by incorporating additional sensory components, allows for a more accurate recapitulation of *in vivo* functionality and serves as a better representative platform for investigating prosthetic design, neuromuscular diseases and understanding the mechanism of action for relevant drugs and their targets^16,17^.

The stretch reflex arc is the physiological system that regulates skeletal muscle movement and tension. This arc can be broken down into two primary components: the efferent and afferent domains. The efferent domain regulates mechanical actuation, muscle tension and relaxation through extrafusal fibers^18^. The regulatory control of tension and contraction is mediated by innervating α-motoneurons. Embedded within the extrafusal fiber tissue are muscle spindles, the sensory organ for afferent (sensory) signals to regulate muscle function^18^. The spindles are composed of intrafusal fibers encapsulated in collagen and innervated by both sensory and gamma-motoneurons (γ-MNs)^18–20^. The primary role of intrafusal fibers is the detection of the magnitude and speed of stretch or flexion of the muscle and the position of the limbs, or proprioception^18,19^. Upon a change in muscle tension, the muscle spindles send signals through afferent sensory neurons which are relayed to neurons within the spinal cord. Motoneurons receiving afferent information can then signal to intrafusal or extrafusal fibers to relax or contract in response to sensory input^18^. With the afferent sensory feedback, the reflex arc acts as an automated closed loop so that voluntary movement can be achieved accurately and properly.

Several types of neurons innervate both the extrafusal and intrafusal fibers of the muscle tissue. The extrafusal fibers are primarily innervated by α motoneurons while intrafusal fibers are innervated by both sensory neurons and γ-MNs^18^. The Type Ia sensory neurons innervate the intrafusal fibers with annulospiral wrappings around the equatorial region and Type II sensory neurons employ flower spray endings towards the peripheral ends of the fiber. γ-MNs innervate the peripheral ends of the intrafusal fibers via flower-spray endings as well. The peripheral ends of the intrafusal fibers slowly relax or contract under the control of γ-MNs. These γ-MNs modulate the tension, sensitivity, and length of the intrafusal fibers so muscle spindles can maintain constant sensitivity during dynamic muscle action and prevent overextension, which can lead to undue stress on the muscle as well as tendon and joint damage^19^. Unlike α motoneurons, γ-MNs do not directly induce muscle tissue contraction or relaxation but modulate the sensitivity of the muscle spindle instead^18^, which then modifies the activity of α-motoneurons (α-MN) and subsequent muscle contraction. Human-based *in vitro* models for the innervation of extrafusal fibers by α-MNs and intrafusal fibers by proprioceptive sensory neurons have been established, but an *in vitro* model for the innervation of intrafusal fibers by γ-MNs has yet to be developed, despite being crucial for proper function of the neuromuscular system. Development and incorporation of this γ-motoneuron - intrafusal fiber system into the reflex arc would provide a better platform for the study of neuromuscular development, prosthetic design, relevant diseases and the evaluation of potential drug candidates.

Investigations surrounding γ-MN interactions have been sparse due to the nature of the cell types involved, and the lack of *in vitro* studies demonstrating this particular cellular interaction, especially with human cells. Although γ-MNs have a specific function in the neuromuscular system, their developmental similarity to α-MNs has made it difficult to identify them for study. γ-MNs originate in the ventral spinal cord along with three subtypes of α-MNs^18,19^. Recently, molecular markers have become available for identifying γ-MNs in co-culture with α-MNs from murine tissue^21–26^. However, functional data of postnatal *in vitro* γ-MNs has yet to be observed and no human systems have been studied.

Utilization of human reflex arc test platforms with the inclusion of γ-MNs could allow for more appropriate analysis of neuromuscular diseases, particularly in relation to proprioceptive function. This is specifically applicable for Spinal Muscular Atrophy (SMA) research. The mechanism for SMA progression in murine models indicated a difference in motoneuron subtype (α versus γ) survival rates compared to analysis of human cells^27^. These data imply that animal models may be insufficient to recapitulate the human form of the disease and more accurate, reproducible, and modular models are needed to fully understand the mechanisms of disease onset and progression. There is also a distinct possibility that this circuit can be involved in neuropathic pain in some cases^28^ and a model system that could investigate this possibility should significantly advance our understandings in this field. In this study, we aimed to develop a de novo defined human-based functional *in vitro* fusimotor system in which the innervation of intrafusal fibers by γ-MNs was evaluated by both immunocytochemical and electrophysiological approaches.

## Results

### Morphological Analysis

**Figure 1:**
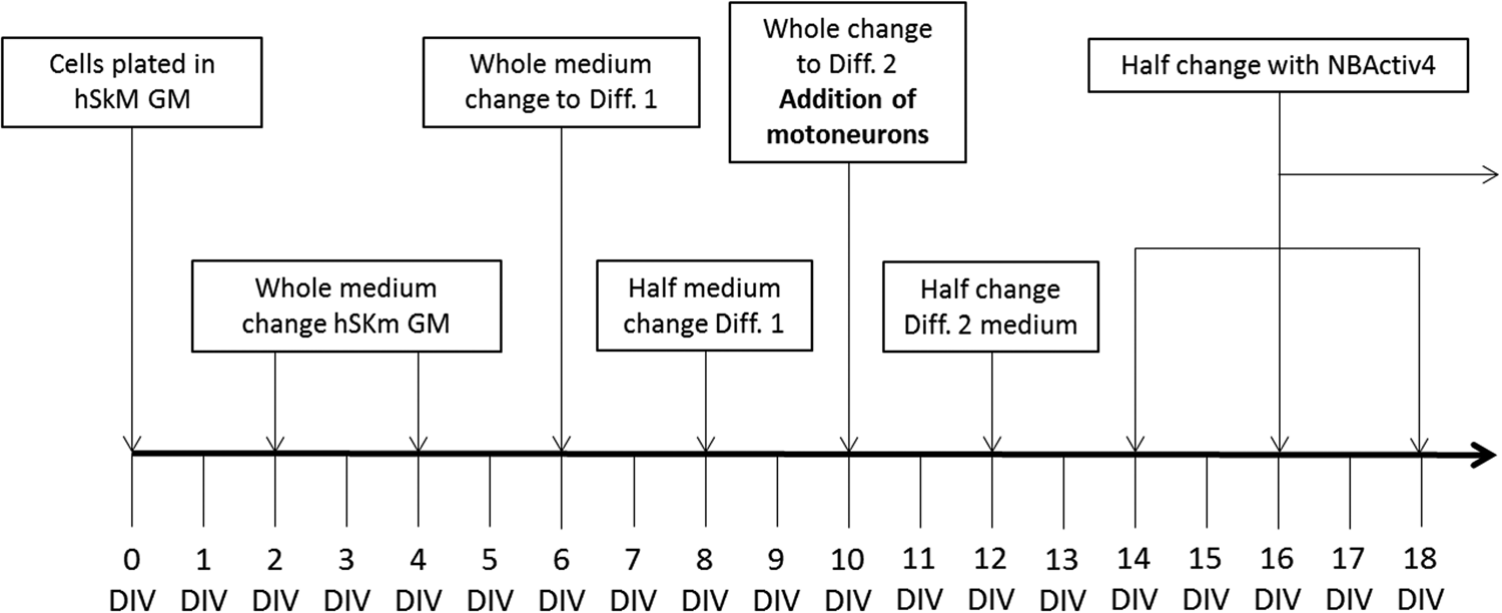
The cell culture scheme for the differentiation of the human intrafusal fibers derived from satellite cells. MNs added on day 10 of the culture were subjected to 2–10 DIV differentiation.

**Figure 2:**
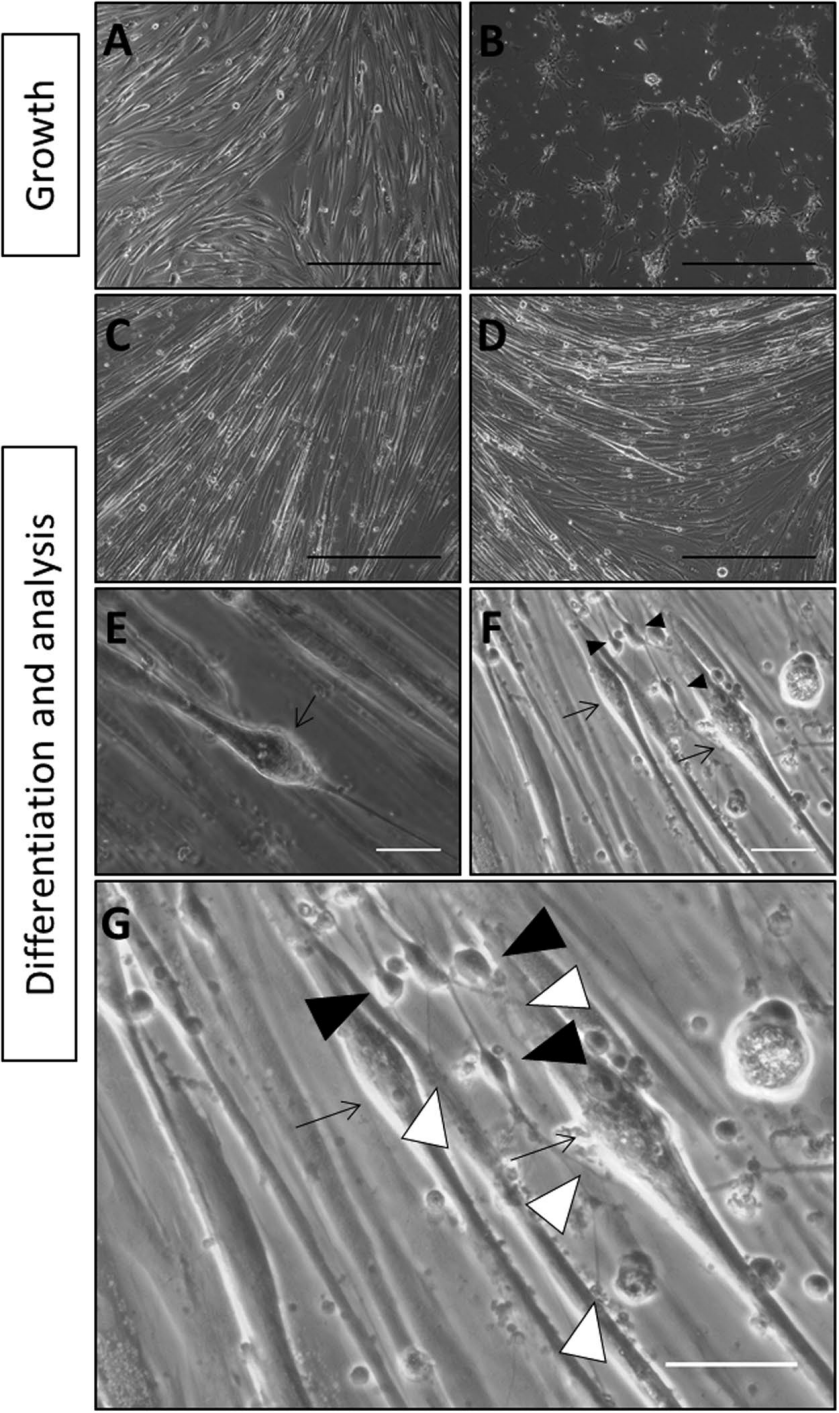
Phase contrast analysis of the human skeletal muscle and motoneurons. **(A)** Human muscle at 4 DIV. (**B)** Human MNs at 4 DIV. (**C** and **D)** Human muscle cultured without **(C)** and with **(D)** MNs at 17 DIV**. (E** and **F)** Morphological confirmation of intrafusal fibers in both muscle only **(E)** and muscle and MNs co-cultures **(F)**. (**G)** An enlarged view of image F to demonstrate the contacts of axon terminals with intrafusal fibers. Intrafusal fibers are indicated with **arrows** and MNs are indicated with black arrowheads. Neuromuscular contacts are indicated with white arrowheads. Black scale bars are 500 µM. White scale bars are 50 µM.

Human intrafusal fibers were co-cultured with human motoneurons (MNs) on trimethoxysilylpropyldiethylenetriamine (DETA) coated glass coverslips in serum-free medium. This surface modification has been used as a means of providing a surface amenable for the proliferation and maturation of an array of cell types for long term experimentation^7,9–12,14,15,29–37^. Figure 1 indicates the protocol for cell expansion and differentiation for developing the co-culture and more detail can be found in Guo *et al*.^15^ and Guo *et al*.^34^. Cellular proliferation and differentiation of intrafusal fibers and neurons were temporally monitored via phase contrast microscopy and assessed via morphological analysis as shown in Fig. 2. Intrafusal fibers and neurons were identified in the culture by their unique morphological features. Chain fibers have long segments of aligned nuclei in the equatorial region and bag fibers have a large cluster of nuclei in the center of the fiber, which tapers down to thin myotube endings used to anchor both ends of the elongated cell within the spindle *in vivo* ^38^. Phase contrast images demonstrated both chain and bag morphologies of intrafusal fibers. Direct physical contacts observed morphologically between intrafusal fibers and neurons indicated possible intrafusal fiber innervation by MNs (Fig. 2F–G).

### Immunocytochemical Analysis

**Figure 3:**
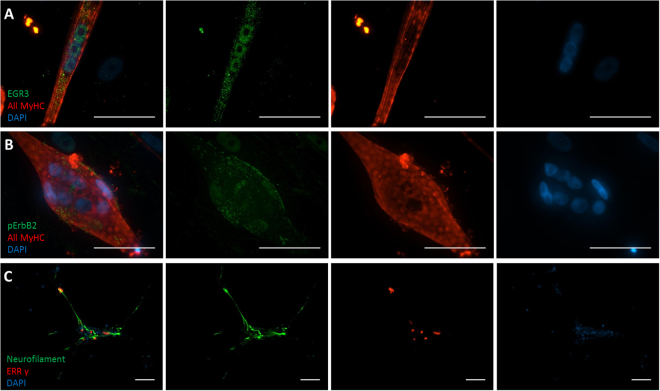
Immunocytochemical analysis of intrafusal fibers and γ-MNs. (**A** and **B)** Intrafusal fibers were identified with the antibodies against EGR3 **(A)** and pErbB2 **(B)**. Myotubes were visualized with the antibody against All Myosin Heavy Chains, and nuclei were identified by DAPI **(A** and **B)**. A sample image of a chain fiber is shown in panel A and bag fiber in panel B. (**C)** Motoneuron cultures were stained with Neurofilament and γ-MNs were additionally stained with an antibody against ERRγ. Scale bars are 50 µM.

After maturation, at approximately day 15 of the human muscle and MN co-culture, cells were immunostained utilizing the molecular markers for both intrafusal fibers and γ-MNs (Table 1). The general marker used to identify muscle fibers in the cultures was All myosin heavy chain (AllMyHC). Neurofilament, a neuronal cytoskeleton marker, was used to non-specifically identify neurons. Intrafusal fibers were identified using pERB2 and EGR3. ErbB2 is a tyrosine kinase receptor for neuregulin, a molecule released by neurons as part of the intrafusal induction mechanism during differentiation^39^. Upon the binding of neuregulin to ERB2, the receptor becomes phosphorylated (pErbB2)^40^, leading to an increase in the transcription factor EGR3, which initiates the expression of genes for intrafusal differentiation^41^. Immunocytochemistry indicated positive identification of both bag and chain intrafusal fibers (Fig. 3).

**Table 1:** Listing of primary antibodies used to characterize γ-motoneurons, intrafusal fibers and the co-cultures.

Antibody	Target	Host Species	Dilution/Concentration	Source	Catalog number
All MyHC	All muscle	Mouse	1:10	Developmental studies hybridoma bank	A4.1025
Phospho-erbB- 2	Intrafusal fibers, activated NRG receptors	Rabbit	2 ug/ml	Millipore	06–229
EGR3	Intrafusal fibers, transcription factor	Rabbit	1:100	SantaCruz Biotechnology	SC-191
ERRγ	γ-MNs	Mouse	1:100	Perseus Proteomics	PD-H6812–00
α-Bungarotoxin 488	Acetylcholine receptors	N/A	1:100	Thermo Fisher	B13422
Neurofilament	Neuronal cytoskeleton	Chicken	1:1000	Millipore	AB5539

**Figure 4:**
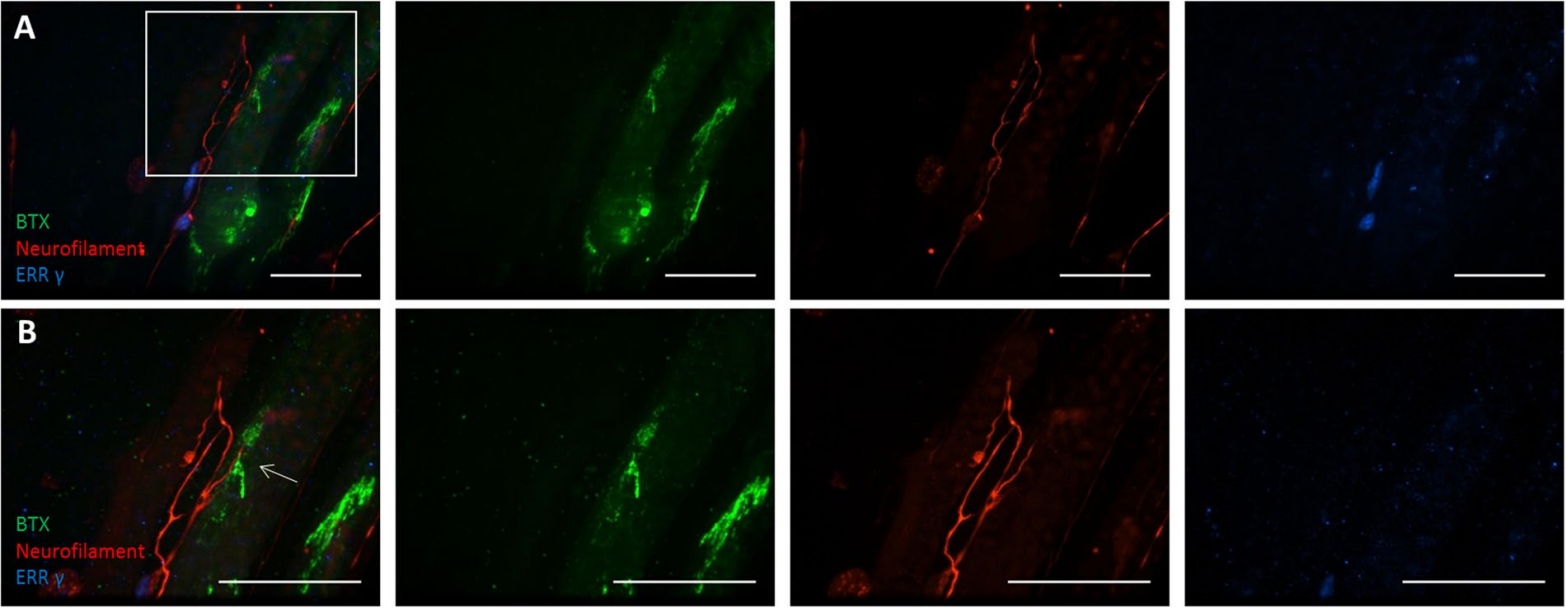
Immunocytochemical analysis of intrafusal fibers and motoneurons co-cultures. (**A)** Co-cultures were stained with Bungarotoxin (BTX), Neurofilament, and ERRγ. (**B)** High definition image for the boxed area in **(B)** indicates a close apposition of an axonal terminal from a γ-MN with BTX-488 patches (white arrow). Scale bars are 50 µM.

Very few markers have been reported for γ-MNs, but ERRγ (27) was found to be the most robust. Other markers were evaluated, including Wnt7a (marker for γ -MNs) and NeuN (marker for α-MNs), but did not have as robust and repeatable of a signal as ERRγ (see Supplementary Figure 1). γ-MNs were recognized by positive immunostaining for ERRγ and the axonal terminals were visualized with neurofilament (Fig. 4A). Since the cultures used have a mixed population, γ-MNs were quantified in a motoneuron preparation and it was found to have 3.63% γ-MNs (±0.86%). Previous work has shown these cultures contain about 20% of motoneurons^35^ so the MN culture consisted of about 18% γ-MNs in the culture. Recent literature from *in vivo* animal studies has also shown that γ-MN innervation of intrafusal fibers is an acetylcholine (ACh)-based neuromuscular interaction^42^. In order to detect the possible innervation of intrafusal fibers by γ-MNs in the co-culture, intrafusal fibers were visualized by fluorescently-labeled bungarotoxin (BTX), a toxin that specifically binds to ACh receptors (Fig. 4). The close apposition of γ-MN axonal terminals or soma with the ACh receptor -positive intrafusal fibers were frequently observed under high definition confocal microscopy, demonstrating immunocytochemical evidence for the potential innervation of intrafusal fibers by γ-MNs. These interactions were quantified as 53.4% (±7.59%) of all neuromuscular appositions. Considering the low percentage of γ-MNs in the motoneuron only culture, interactions between intrafusal fibers and γ-MNs appear to be enhanced over interactions between intrafusal fibers and other neuron types in the culture. This may imply a selective mechanism to promote NMJs between γ-MNs and intrafusal fibers.

### RNA Expression Analysis

**Figure 5:**
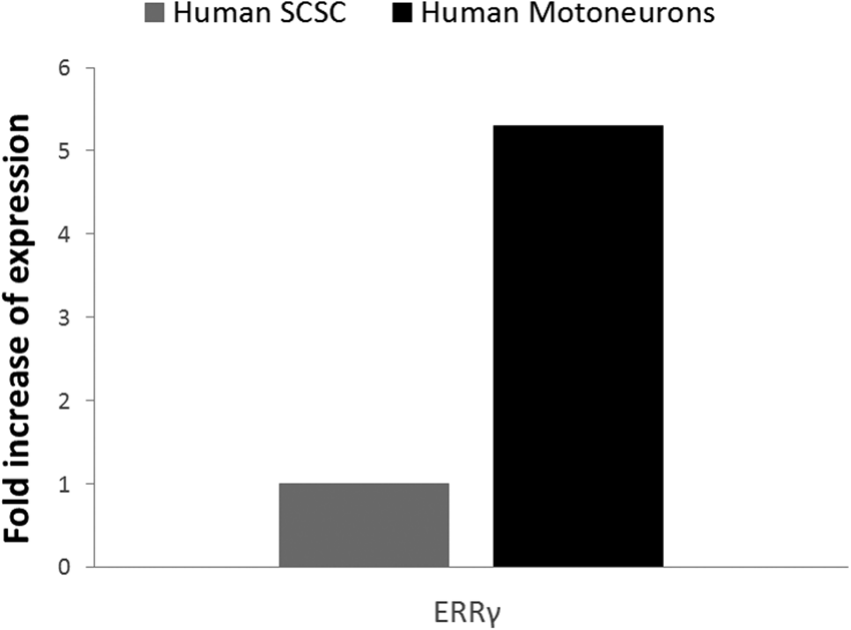
Quantitative PCR: Quantification of ERRγ in the motoneuron culture compared to that in their undifferentiated precursors, human SCSCs.

Immunocytochemistry results for γ-MN percentage in the MN cultures were confirmed by quantitative PCR analysis. RNA expression of ERRγ was examined in human motoneuron cultures, utilizing the undifferentiated neural progenitors (human spinal cord stem cells, or SCSCs) as the control. There was found to be a 5.3 fold increase in ERRγ expression in differentiated human motoneurons compared to the control (Fig. 5). This confirmed the increase in expression levels of the γ-MN specific marker ERRγ in the immunocytochemical data, demonstrating the presence of γ-MNs in the heterogeneous culture.

### Electrophysiological Analysis

**Figure 6:**
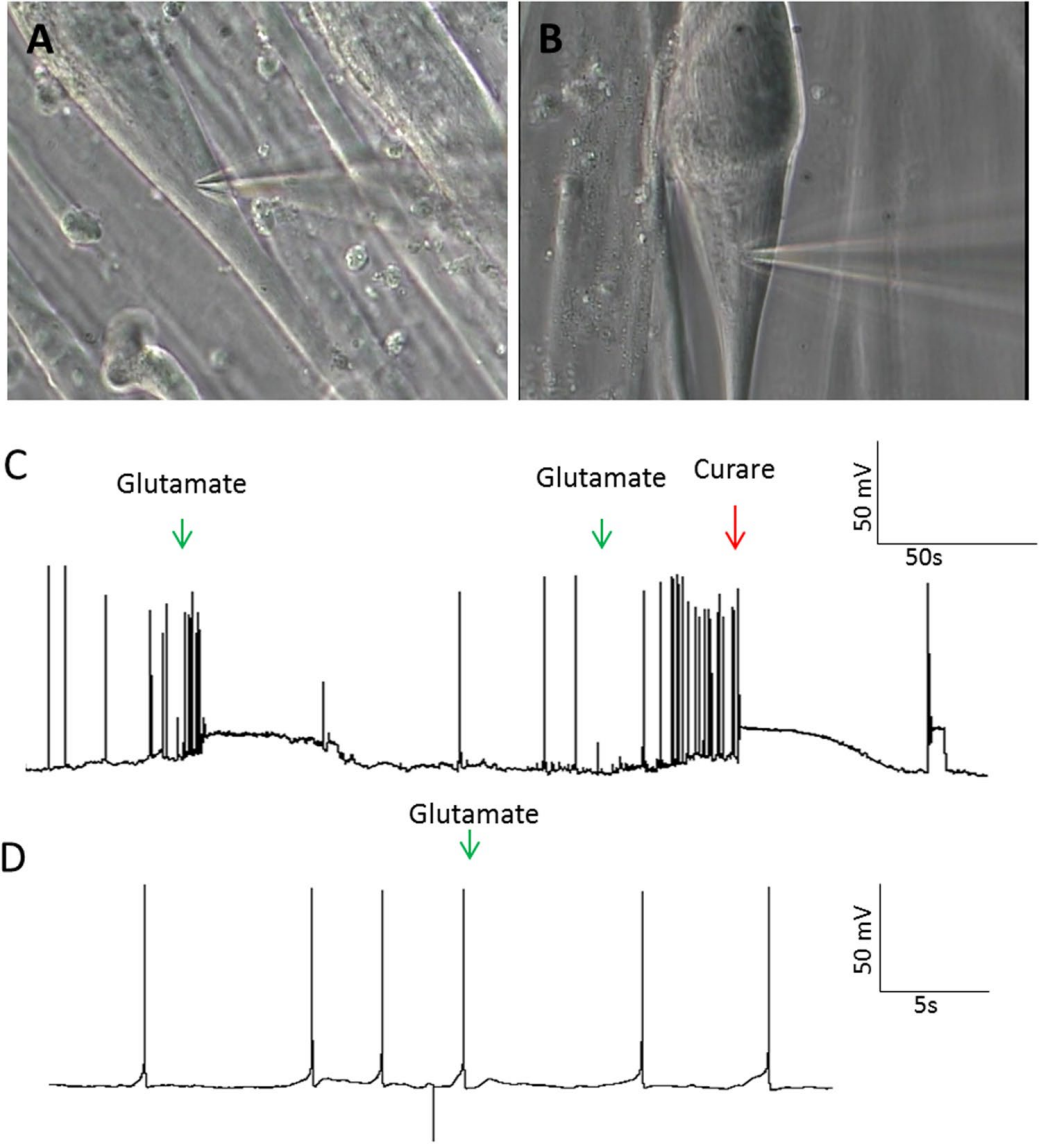
Patch-clamp analysis of intrafusal fibers. (**A,B)** Representative bright field microscopy of patched intrafusal cells in motoneuron-muscle co-cultures **(A)** and muscle only controls **(B)**. (**C,D)** Gap-free recordings from patched intrafusal fibers in motoneuron–muscle co-cultures **(C)** and muscle only controls **(D)**. Addition of glutamate (marked with green arrows) in the co-culture elicited increased activity and addition of curare (marked with red arrow) terminated activity **(C)** but no activity change was induced by either of them in intrafusal fibers in the muscle culture alone **(D)**.

**Figure 7:**
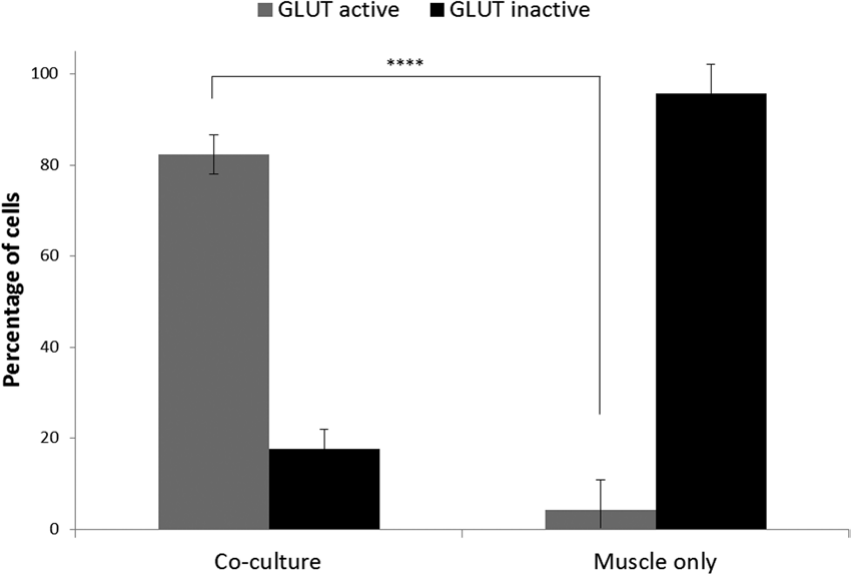
Statistical analysis. Percentages of glutamate responding and glutamate nonresponding intrafusal fibers in muscle-motoneuron co-culture conditions and muscle only conditions. P value = 2.17e-9.

The immunocytochemical evidence strongly suggested that the stimulation of γ-MNs would lead to electrophysiological activity in the intrafusal fibers. In order to determine if the innervations identified immunocytochemically were functional, patch-clamp electrophysiological recordings were performed on intrafusal fibers (15–30 days in vitro (DIV)). Glutamate is an excitatory neurotransmitter that has been previously used to stimulate neurons in co-cultures with muscle without directly initiating myotube contraction^14^. The electrophysiological response to the addition of glutamate, both with and without MNs in the culture, was recorded from intrafusal fibers, identified by their morphological characteristics, in the co-culture (Fig. 6A,B). Theoretically, the intrafusal fiber, if innervated, should be excited upon glutamate addition, while those not innervated should not be affected. As indicated, bursts of myotube action potentials (APs) were induced in multiple intrafusal fibers upon glutamate addition, suggesting innervation by γ-MNs (Fig. 6C). To confirm that the increased AP firing was initiated by ACh-mediated innervation, a blocking agent for ACh mediated synaptic transmission, curare, was applied after increased firing was initiated by glutamate treatment^14^. Immediate cessation of electrophysiological activity was observed (Fig. 6D). This demonstrated the functional innervation of the intrafusal fibers by γ-MNs. As a control, the same experiment was performed on intrafusal fibers in the absence of MNs where no significant change of activity was detected. Quantification of the co-cultures indicated that 28 out of 34 intrafusal fibers were excited upon glutamate addition, while only 1 out of 23 muscle-only controls were excited (p value = 0.2.17e-9) (Fig. 7). The few intrafusal fibers not excited in the co-culture may not have been innervated, resulting in a lack of response to MN activity. This data confirms not only the formation of functional synapses but also demonstrates a high percentage of γ-MN innervation of the intrafusal fibers under these co-culture conditions.

## Discussion

We have developed a human-based *in vitro* fusimotor system in which functional innervation of intrafusal fibers by γ-MNs was demonstrated by morphological, immunocytochemical and electrophysiological analysis. Neither the culture of human γ-MNs nor their innervation by intrafusal fibers has previously been shown *in vitro*. The recapitulation of the spindle fusimotor circuit *in vitro* with human cells provides a defined model to investigate the physiology of this circuit, which is otherwise extremely difficult to observe for human systems. The use of MNs and muscle derived from iPSCs also offers the possibility for their incorporation into human-on-a-chip systems designed for the investigation of patient specific neuromuscular diseases and deficits.

The functional sensory circuits, especially human based systems, have previously not been extensively investigated *in vitro* and γ-MN identification and functional innervation had not been demonstrated with human cells at all. Here, a co-culture of human intrafusal fibers and human MNs was established to evaluate the interactions between these two important cell types from the sensory portion of the reflex arc. Initial morphological analysis via phase contrast microscopy indicated the two cell types were compatible in a serum-free defined system and formed close physical contacts suggesting synaptic interactions. Immunocytochemical analysis confirmed the identity of the cell types in these intercellular interactions. Intrafusal fibers stained positive for pERB2 and EGR3, while the γ-MNs were identified by immunostaining with a γ-MN specific marker ERRγ^19,21,25^.

Electrophysiological analysis confirmed functional interactions between the γ-MNs and intrafusal fibers. γ-MNs were stimulated using the neurotransmitter glutamate. The majority of intrafusal fibers in co-culture demonstrated increased excitation upon the application of glutamate (28 out of 34), while only one intrafusal fiber in the monoculture responded. Furthermore, the glutamate-induced responses were terminated by the application of curare, a competitive antagonist of acetylcholine receptors. These data indicate functional innervation of human intrafusal fibers by human γ-MNs in our defined *in vitro* system.

It should be pointed out that a single intrafusal fiber in monoculture conditions exhibited AP firing upon exposure to glutamate. The presence of glutamate receptors on muscle fibers is known and has been researched^43,44^. Multiple mechanisms have been proposed regarding the function of glutamate receptors in muscle tissue, but none of which correspond to direct action potential generation^45–50^. Actually, a similar response was recorded from another intrafusal fiber in a muscle-only culture by adding media instead of glutamate (data not shown), indicating the addition event itself could non-specifically induce muscle excitation, although very rarely. To further confirm the muscle was not directly excited by glutamate in our system, glutamate dosage experiments (data not shown) were performed on muscle only cultures to evaluate the responsiveness of intrafusal fibers to increasing concentrations of glutamate. The lack of glutamate mediated electrical activity in these cultures, even at concentrations far higher than used in experiments reported here, demonstrates the lack of direct response to glutamate. This evidence confirms that the single cell responsive to glutamate was not the result of a glutamate induced electrophysiological mechanism in this human model.

Study of the entire functional reflex arc is essential to understand and further investigate many neuromuscular disorders, predominantly Amyotrophic Lateral Sclerosis (ALS) and Spinal Muscular Atrophy (SMA). In both reports the sparing of γ-MNs were reported in mice models as well as peripheral neuropathies^27,51^. Most existing *in vitro* systems used to study these diseases only investigate the motor perspective of neuromuscular interactions. However, utilization and especially integration of sensory components is essential to investigate complicated neuromuscular diseases by the inclusion of afferent mechanisms. Additionally, the sensory portion of the reflex arc has been of interest to the field of pain research^28^. Drug discovery has been moving towards more repeatable and high-throughput organ-on-a-chip *in vitro* systems recently for use in preclinical compound evaluation to improve drug discovery efficiency^1–8^. These systems provide a highly controllable and repeatable platform that can be tailored to more human-specific diseases by utilizing patient-derived iPSCs. Incorporation of γ-MN and intrafusal interactions to such systems will allow for a more complete and refined model for simulating neuromuscular diseases and testing a multitude of drug response variables.

## Materials and Methods

### Surface modification

18 mm round glass coverslips (VWR, 48380-046) were cleaned and functionalized by exposure to an oxygen plasma generated by a Harrick plasma cleaner (model PDC-32G) utilizing high purity oxygen gas. The surfaces were then submersed in a reaction solution of 1% v/v of trimethoxysilylpropyldiethylenetriamine (DETA, United Chemical Technologies, Inc., T2910-KG) in dry toluene (VWR, BDH1151-4LG) and heated to just below the boiling point of toluene over a period of 30 minutes. The reaction vessel was removed from heat and allowed to cool for 30 minutes, and then rinsed in 3 serial toluene baths. Next, the surfaces were placed in dry toluene and heated to just below the boiling point of toluene over a period of 30 minutes. After the second heating step, the surfaces were cured in an oven at approximately 110 °C overnight (~15 hr). Derivatized surfaces were characterized by contact angle goniometry, with a 5 µL droplet of water, and by X-ray photoelectron spectroscopy using a Thermo Scientific ESCALAB 220i-XL instrument with aluminum Kα X-rays and a 90° take-off angle^52,53^.

### Cell Culture

***Human intrafusal fibers*** were differentiated from human satellite cells provided as a gift from Dr. Herman Vandenburgh. Human skeletal muscle stem cells (hSKM SCs)/progenitors were isolated and proliferated as described in Thorrez *et al*.^54^. Briefly, the primary human skeletal muscle cells (hSKMs) were isolated by needle biopsy^55^ and expanded in the myoblast growth medium (MGM; SkGM (Cambrex Bio Science, Walkersville, MD) plus 15% (v/v) fetal bovine serum. Biopsies were performed on adult volunteers according to procedures approved by the Institutional Clinical Review Board of the Miriam Hospital and were performed in accordance with the relevant guidelines and regulations. All samples, from the study participation and publication of images, were obtained with informed consent and de-identified before being sent to UCF. Cell preparations averaged 70% myogenic content based on desmin-positive staining^56^. The differentiation protocol was adapted from our protocol for extrafusal fiber differentiation by inclusion of specific factors to facilitate intrafusal differentiation^15^. Specifically, thawed satellite cells from liquid nitrogen were plated onto glass coverslips at a density of 100 cells per mm^2^. The cells were given a whole medium change of human skeletal growth medium every two days until confluent. Upon confluence, the medium was fully switched to differentiation 1 medium (DMEM (Invitrogen 11775-040), Insulin (Sigma I9278) at 10 µg/ml, BSA (Sigma A9418) at 500 µg/ml, and EGF (Invitrogen 13247-051) at 10 ng/ml)^34^ and fed every 2 days for 4 days. At this point, the medium was switched to differentiation 2 medium (described in detail in Guo *et al*.^15^) and MNs were added to co-cultures at 50 cells per mm^2^. The cells were fed every two days with differentiation 2 medium and maintained for four days. After this point, the medium was given a half change with NBActive4 differentiation medium. The cells were maintained in this medium for the remainder of the culture and fed every two days until 15–30 DIV total when they were analyzed via electrophysiology or fixed for immunocytochemical analysis.

***Human Motoneurons*** were differentiated from spinal cord stem cells (SCSCs) NSI566RSC according to a previous report^9^. Specifically, thawed SCSC cells (0.5 E6) from liquid nitrogen were plated onto a T-25 cell culture flask coated with Poly-D-Lysine. The cells were supplemented with bFGF every day and fed every other day with N2B medium. Upon confluence, the cells were trypsinized with 0.05% Trypsin and the reaction was inhibited with the addition of 0.2% Trypsin inhibitor (final 0.05%). The cells were replated onto a permanox dish (Diameter 60 mm) coated with Poly-D-Lysine and fibronectin. Cells were initially plated in priming medium and fed on day 2 of culture with a half change of medium. On day 4 the medium was half changed with human MN medium. The cells were maintained in the permanox dish for about 10 days. At this point cells were again trypsinized and purified via an Optiprep concentration gradient (Optiprep diluted to 30% [v/v] utilizing Neurobasal with and without phenol red (ThermoFisher Cat#21103-049 and Cat#12348-017, respectively) and then made to 9.5, 7.0, 6.0, and 5.5% for fractions 1–4, bottom to top sequentially [v/v] in Neurobasal, with fractions 1 and 3 containing phenol red and fractions 2 and 4 lacking phenol red for optimizing visualization of the fractions). The cells were plated into DETA coated glass coverslips for less than 10 days before being trypsinized (as described above) and replated onto either muscle cultures or control DETA coated glass coverslips. At this point the neurons either underwent exposure to intrafusal differentiation media (co-cultures) or were maintained in human MN medium (monocultures).

### Immunocytochemistry

Cells on coverslips were fixed with 4% paraformaldehyde diluted in PBS solution for 15 minutes, then rinsed with PBS (phosphate buffered saline) for 5, 10, and 15 minutes and stored until staining in PBS. Cells were permeabilized with 0.1% Triton X-100 for 15 minutes then incubated for 1–2 hours at room temperature in blocking buffer (5% Donkey serum +0.5% BSA in PBS) to prevent nonspecific binding. Fixed and permeabilized cells were incubated overnight with primary antibodies (diluted in blocking buffer) at 4 °C. Antibodies used and their concentrations are listed in Table 1. The cells were rinsed with PBS for 5 minutes, 0.01% Trition X-100 for 10 minutes and PBS for 15 minutes, and then subjected to incubation with the secondary antibodies (1:250 diluted in blocking buffer) for 1–2 hours at room temperature. The cells were then rinsed with PBS for 5 minutes, 0.01% Trition X-100 for 10 minutes and PBS for 15 minutes and mounted onto glass slides using ProLong Gold Antifade Mountant with DAPI (Thermo FisherP36931) or Vectashield mounting medium for fluorescence (Vector laboratories, Burlingame, CA) and imaged using a Zeiss LSM 510 confocal microscope.

### Quantitative polymerase chain reaction

Human motoneurons and human spinal cord stem cells were harvested for RNA extraction at less than 10 days *in vitro*. The RNA extraction was done utilizing the Aurum™ Total RNA Mini Kit (Biorad 7326820). RT-PCR was performed using the iTaq™ Universal SYBR® Green One-Step Kit (Biorad 1725150). Reaction volumes was 25 µl total (12.5 µl 2X SYBR^®^ Green reaction mix, 0.75 µl forward primer at 10 µM, 0.75 reverse primer at 10 µM, 1.0 µl iTaq DNA polymerase, 10 ng RNA template, and enough water to fill the remaining volume). Reactions were incubated at 50 °C for 10 minutes, 95 °C for 5 minutes, 95 °C for 10 seconds and 55 °C for 30 seconds cycled 40 times, and underwent melt curve analysis (95 °C for 1 minute, 55 °C for 1 minute, and 55 °C to 95 °C for 80 cycles for 10 seconds, increasing 0.5 °C each cycle). ERRγ (Forward: 5′-AGGAAAACCTATGGGGAATG-3′; Reverse: 5′-GGAGCAAATGAAATGTGGGTG-3′) expression was evaluated along with β-actin (Forward: 5′-CCCCATTGAACACGGCATTG-3′; Reverse:5′-ACGACCAGAGGCATACAGG-3′) as a positive control and template free β-actin reactions as negative controls. Samples were run in triplicate along with β-actin positive controls and template free negative controls. An MJ mini thermal cycler (Biorad) and Opticon Monitor software (Biorad) were used. Expression levels were corrected with β-actin levels.

### Electrophysiology

Whole-cell patch clamp recordings were performed in a recording chamber on the stage of an upright microscope (Axioscope FS2, Carl Zeiss, Göttingen, Germany). The patch-clamp recording chamber was filled with the same medium as utilized for cell culture. The intracellular solution composition was (in mM): K-gluconate 140, NaCl 4, CaCl_2_ 0.5, MgCl_2_ 1, EGTA 1, HEPES acid 5, HEPES base 5, Na_2_ATP 5. Patch pipettes were prepared (borosilicate glass, BF150-86-10; Sutter, Novato, CA) with a Sutter P97 pipette puller. Pipette resistance was 4–10 MΩ for intracellular patch clamp recordings. Experiments were performed with a Multi clamp 700B (Axon Instruments, Foster City, CA, USA) amplifier. Signals were filtered at 2 kHz and digitized at 20 kHz with an Axon Digidata 1322 A interface. Action potentials were recorded in current-clamp mode under gap free conditions at zero holding potential. The series resistance was in the range of 5–10 MΩ and was compensated by 60% on-line. Leak currents were subtracted using a standard P/4 protocol. Before seals were established on the cells, offset potentials were nulled. Capacitance subtraction was used in all recordings. All intrafusal fibers chosen in co-culture conditions were in proximity to MNs identified via morphological analysis. To test whether the patched intrafusal fiber was innervated, glutamate was added in 30 ul doses of 200 mM to the extracellular solution in the vicinity of the patched intrafusal fiber. When excited, the MNs would excite innervated muscle fibers and the excitation was recorded. Once repetitive firing was consistent, curare was added in 30 ul doses of 500 uM to the extracellular solution.

### Statistical analysis

The proportion of active intrafusal fibers that showed electrical activity in response to glutamate was compared between muscle only and co-cultured conditions by categorizing each patched cell as responsive or unresponsive and organized into a contingency table, followed by statistical analysis via Fisher’s Exact Test (α = 0.05). 57 cells were patched across multiple coverslips and platings. The standard error of the mean for each proportion was calculated for the binomial distribution. γ-MN quantification data was taken from 20 view fields from each of three separate coverslips spanning over two separate platings. For γ-motoneuron- intrafusal fiber interaction quantification, two separate co-culture coverslips from two separate platings were stained with ERRγ, BTX488, and neurofilament. Intrafusal fibers positive for BTX488 staining that had contact with neuronal processes positive for neurofilament that lead to an identifiable soma were counted. Standard deviations were calculated and expressed as percent error.

## Electronic supplementary material

Supplementary information
