# Supplementary material for: Functional analysis of human intrafusal fiber innervation by human γ-motoneurons
Source: Sci Rep. 2017 Dec 8;7:17202. doi: 10.1038/s41598-017-17382-2 (PMC5722897; doi:10.1038/s41598-017-17382-2)
Supplement: Supplementary file 1 — Supplementary information [file 41598_2017_17382_MOESM1_ESM.pdf]

Supplementary Information

Functional analysis of human intrafusal fiber innervation by human γ-motoneurons.

Authors: A. Colón, X. Guo, N. Akanda, Y. Cai and J.J. Hickman*

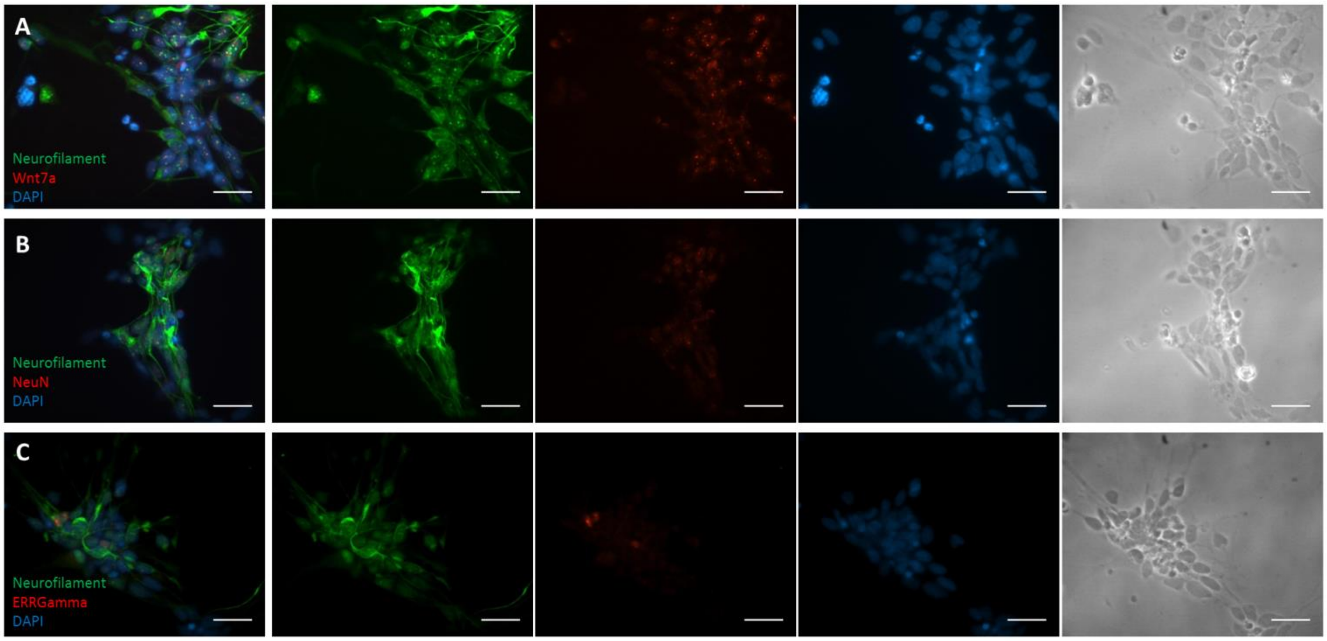

Supplementary Figure 1. Immunocytochemical analysis of motoneuron only cultures. Markers for Wnt7a (Abcam ab100792) (A) NeuN (Millipore MAB377) (B) and ERRGamma (C) immunocytochemistry in motoneuron only controls. Scale bars are 50 µM.
